# Determining infrastructure requirements for an air taxi service at Cologne Bonn Airport

**DOI:** 10.1007/s13272-021-00544-4

**Published:** 2021-08-27

**Authors:** Eva Feldhoff, Gonçalo Soares Roque

**Affiliations:** Institute of Transport Science, RWTH Aachen, Mies-van-der-Rohe-Straße 1, 52074 Aachen, Germany

**Keywords:** Urban air mobility, Air taxi services, eVTOL, Vertiport, Infrastructure capacity, Cologne Bonn Airport

## Abstract

The worldwide increasing population density in major urban centres poses great challenges for transportation systems. Air taxi services could be a solution to this growing problem by bringing the existing transportation system to the three dimensional space. This paper analyzes the challenges and requirements of developing a vertiport intended for the use of air taxis at Cologne Bonn Airport. This research was conducted with the information available at the time of writing, for which a basis scenario is defined for the Cologne Bonn Airport where important aspects of an air taxi service are determined such as passenger demand and possible vehicles. The main aspects analysed were the requirements on the vertiport infrastructure and its location, as well as the requirements on passenger processing. For the defined basis scenario, results show that the preferential locations to develop a vertiport at Cologne Bonn Airport are the roof top levels of parking garages *P2* and *P3*. Furthermore, it is shown that given the estimated passenger demand, a very high utilization factor of the defined infrastructure is to be expected. This paper provides a starting point for the development of an air taxi service at Cologne Bonn Airport. Further research is needed in key issues such as the financial aspects of an air taxi service, its integration into the current operating scenario of the Cologne Bonn Airport and the approval process for an air taxi service and the vertiport itself.

## Introduction

A considerable number of air taxi projects are under development worldwide, with individual developers pursuing different concepts and strategies. Generally, the integration of this new technology into the existing infrastructure and public transport network is increasingly being discussed in society and politics. The aim is to help reduce the increase in traffic congestion, special in inner cities and large urban centres. Furthermore, most of the vehicles in development focus on environmental friendly propulsion methods (generally either electric or hybrid propulsion), for which the introduction of this technology promises not only to help unburden the current traffic network, but also to do it in an environmental friendly way without further deteriorating the climate.

In the greater Cologne Bonn area and the entire airport region, congestion of the ground-based transport infrastructure can be observed every day, especially in the road network [1]. The urgent need to improve the local public transport network also requires solutions for the growing demand on individual and flexible intermodal transport connections, especially in the surroundings of international airports such as the Cologne Bonn Airport.

The dynamic development in the field of Urban Air Mobility is shown by studies from different management consultancies such as Porsche Consulting, Roland Berger and Horváth & Partners [2–4]. These focus on estimating the market potential and identifying relevant application scenarios. On the other hand, a large number of publications and ongoing projects for the development of air taxi vehicles can be attributed to both established and newly founded aircraft manufacturers. These include companies such as Airbus, Lilium and Volocopter [5–7]. Generally, research work focusing on the ground infrastructure required for an air taxi service has been of secondary importance in comparison to the topic of vehicle development.

**Fig. 1:**
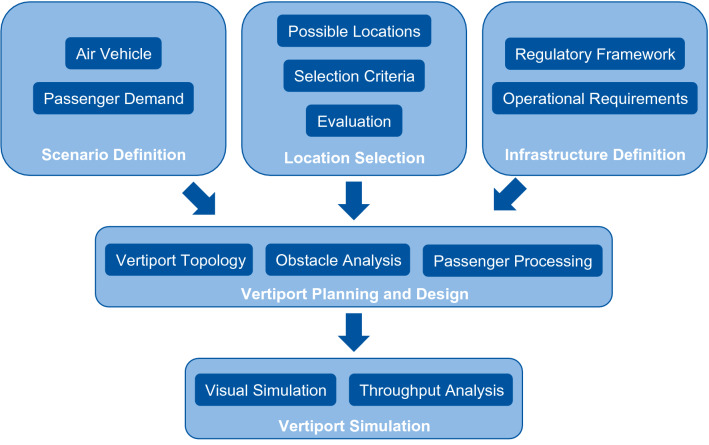
Study methodology flow chart

This study provides clarity on the expected opportunities, challenges and requirements of developing an air taxi infrastructure (so-called vertiport) and integrating an air taxi service into the existing transport network at Cologne Bonn Airport. The focus of this paper is the current situation at the airport, and considers the short to medium term development of infrastructure purposed towards the operation of air taxis. Figure 1 provides an overview of the methodology of this study.

This paper is structured as follows: Sect. 2 presents the Cologne Bonn Airport scenario considered for this work, including passenger demand and air taxi vehicle considerations. “Vertiport infrastructure” discusses the regulatory framework for a vertiport and resulting legal and operational requirements. “Location analysis” provides an overview of possible locations and their assessment. “Passenger processing” indicates required passenger processing components. The simulation analysis of the developed infrastructure and processes is presented in “Vertiport simulation”. Finally, “Conclusion and further work” concludes the main results and gives an outlook for further research steps.

*Note: Statements in this paper regarding traffic volumes refer to the situation in 2019, before the impact of SARS-CoV-2*

## Cologne Bonn Airport scenario

To take into account the local conditions at Cologne Bonn Airport, we first define a scenario that represents the framework in which the vertiport is to be integrated. As one of the main aims of an air taxi service at Cologne Bonn Airport is to expand flexible intermodal transport connections, we focus our work on air taxi services operating at the landside area of the airport. On the other hand, the potential passenger demand as well as suitable air taxi vehicle parameters are considered as key influence factors for further steps. These factors are therefore discussed in the following sections.

### Passenger demand

The estimation of the passenger demand for future air taxi services is very difficult due to the vast number of influencing factors such as socio-economic factors, the quality of the existing transport network and the potential time savings by air taxi services. The detailed investigation of the passenger demand for air taxi services at Cologne Bonn Airport is not within the scope of this work. However, an initial estimation seems reasonable to verify if the demand forms a limiting factor to the air taxi system.

To estimate the potential passenger demand of an air taxi service at Cologne Bonn Airport, we assume that the traffic volume mainly depends on the passenger volume at the airport. The daily traffic volume was calculated by looking at the 30th busiest day of the year, which corresponds to the method of dimensioning airport infrastructure recommended by the International Civil Aviation Organization (ICAO) [8]. This method was applied for the year of 2019. At a future stage, passenger demand growth rates may have to be applied to determine the demand at time of market entry of air taxi services.

The financial structure of air taxi service providers is dependent on a number of factors, including the pursued business model. In this paper, we do not evaluate pricing strategies and customer’s price acceptance. Instead, we estimate the potential passenger volume by looking at transport mode specific changing rates, as the modal split choice includes customer specific preferences and sensitivities. These changeover rates are then applied to the modal split of the Cologne Bonn Airport, as this information is available through passenger surveys. This results in an estimated passenger transportation mode changeover rate of approximately 5%. Although the modal split including air taxi services will highly vary for different local transportation systems, a 5% share of trips by air taxi services lies within a reasonable order of magnitude, compared with estimates of other literature [3, 9]. As the passenger volume of Cologne Bonn Airport summed to 12 million passengers in 2019, this changeover rate would indicate an air taxi service demand of approximately 600,000 passengers per year.

### Air taxi vehicles

The amount of ongoing air taxi vehicle projects shows a high variety of intended use and propulsion concepts. Vehicle characteristics such as largest dimension, seating capacity or flight performance change significantly between different vehicles. Due to the local conditions at Cologne Bonn Airport, we focus only on air taxi vehicles powered by fully electric propulsion technologies, which have the ability of vertical take-off and landing (eVTOL). A comprehensive overview of eVTOLs that are currently certified or under development is given by the websites *eVTOL.news* and *transportUP.com* [10, 11]. To meet the specific requirements at Cologne Bonn Airport, we define several criteria, presented in Table 1, which need to be fulfilled by the eVTOLs. This method provides a selection of relevant eVTOLs for the Cologne Bonn Airport, and can be repeated iteratively, when further developments occur.

**Table 1:** Criteria and choice of considered eVTOLs

Criteria	Relevance	Choice
Vehicle category	Commercial air taxi use	No hover bikes or personal flight devices
Flight control	Visual flight rules (VFR) operations	Manual pilot control interface
Transport capacity	Payload and range requirements	2–5 seats
Project maturity	Certification required for the commercial use	Projects in flight testing

The eVTOLs, which meet the presented criteria, are the following:

- CityAirbus by Airbus Helicopters
- Elroy by Astro Aeronautics
- Passenger Air Vehicle by Aurora Flight Sciences
- Whisper by Electric Aircraft Concept
- S4 by Joby Aviation
- Lilium Jet by Lilium
- Robinson R44 by Tier 1 Engineering
- Volocopter 2X by Volocopter
- Cora by Wisk Aero

## Vertiport infrastructure

### Regulatory framework

To date, there is no regulatory organisation, agency or government, which has published standards or recommendations for the specific case of infrastructure purposed towards the operation of eVTOLs. It can be assumed that specific regulations for the operation and infrastructure of eVTOLs will be developed, based on already existing regulations. Due to the physical characteristics of eVTOLs and their vertical flight capability, the vehicle found to be the most comparable to an eVTOL is the helicopter. Therefore, this study derivers the legal requirements for the development of a vertiport from the existing documents that regulate the design of heliports. These are namely the ICAO *Annex 14, Volume II Heliports* [12] and the ICAO *DOC 9261 Heliport Manual* [13]. It should be noted that due to the technological advances that have been incorporated into the development of eVTOLs, it is expected that their control and navigation capabilities will be at least equivalent, and most likely better, than those of helicopters. Therefore, the method used in this study is rather conservative. The most relevant topics in the adaptation from the regulations for heliports are the requirements for the physical characteristics and the obstacle environment.

### Infrastructure requirements

The infrastructure needed to operate a vertiport is highly dependent on the intended use. It can consist of a single landing and take-off area (so-called Final Approach and Take-Off Area: FATO) where a vehicle can land and take-off, or it can be a whole hub with multiple FATOs, each one supported by multiple stands where passengers can board and deboard the vehicle. Due to the characteristics of the Cologne Bonn Airport, which is already a major multi-modal transportation node, and the potential passenger demand described in “Passenger demand”, it is assumed that the infrastructure needed to support an air taxi service at the airport will eventually have the characteristics of a transport hub. However, the concept of an air taxi is still very much in development, and the entry into operational service of this new method of transportation is certainly going to happen gradually. For these reasons, this study assumes that the infrastructure to support an air taxi service at Cologne Bonn Airport will initially consist of one single FATO, supported by multiple stands. This initial infrastructure is not specifically designed for the estimated passenger demand (“Passenger demand”) and is not expected to handle this demand sufficiently but rather forms the basis for further infrastructure expansions.

#### Regulatory requirements

As explained in “Regulatory framework”, this study assumes that the infrastructure for the operation of eVTOLs will have the same characteristics as a heliport, and be based on the same regulatory framework. The focus is placed foremost on two aspects of this regulatory framework: the physical characteristics and the obstacle environment. The physical characteristics of a vertiport are dependent on the largest overall dimension of the largest eVTOL for which the vertiport is intended. Through an analysis of the considered eVTOLs, listed in “Air taxi vehicles”, the dimension selected is 12 m. The obstacle environment analysis has the objective of identifying, for the various vertiport locations, which existing or planned infrastructures would possibly represent an obstacle to the operation of eVTOLs. This is established through the definition of obstacle limitation surfaces, whose characteristics are dependent on the physical characteristics of the largest eVTOL as well as on the surface slope category. This study assumes the largest overall width also to be 12 m and the use of a category A slope, which is the most stringent of all the slope categories with a slope of 4.5%.

#### Requirements on stands

To understand the surface requirements of the whole vertiport, it is essential to know how many stands are exactly needed to support the single FATO. Two important quantities are required to estimate the number of stands needed: the time that a single eVTOL blocks the FATO during take-off and landing and the time an eVTOL needs after landing to load its batteries, before being able to take-off again. Through discussions with the air navigation service provider in Germany (Deutsche Flugsicherung GmbH), the landing and take-off times are assumed to be the same and equal to 3 min. This means that each eVTOL blocks the FATO for a total of six minutes per landing and take-off cycle, which determines the capacity of the FATO to be 10 aircrafts per hour. An accurate estimation of the time that an eVTOL needs to load its batteries is very hard to achieve, as most vehicles are still in development and the data of each vehicle is normally kept secret until the certification process. Through a combination of expert interviews and publicly available data [14], the average battery loading time is assumed to be 30 min. This restricts the capacity of each stand to two aircrafts per hour. Having estimated these two individual capacities, the number of stands needed to support the single FATO can be calculated through Eq. (1), which gives the result of five stands.

1 $$ {\text{Stands Required}} = {\text{FATO Capacity / Stand Capacity}} $$

However, Eq. (2) developed by Vascik implies additional taxi time and gives the result of nine stands, assuming a taxi time of 1 min [15].

2 $$ {\text{Stands Required}} = {\text{Ceiling}}\left( {\frac{{\text{Turnaround Time}}}{{{\text{max}}\left( {\text{Arrival Time, Departure Time}} \right){\text{ + Taxi Time}}}}} \right){ + 1} $$

We, therefore, consider the result of five stands as minimum requirement that can desirably be extended to the number of nine if the required footprint is available.

### Location selection criteria

Besides the regulatory requirements imposed on a vertiport, a list of criteria, which the location of the vertiport should fulfil, was also defined for the specific case of the Cologne Bonn Airport to facilitate the selection and evaluation of the possible locations. These criteria are defined as follows:

- Passenger accessibility: the site must be easily accessible to arriving and departing passengers from the airport terminals 1 and 2. A frequently used indicator for this criterion is the average walking distance between the individual terminals and the vertiport.
- Obstacle Clearance: the site must be located so that its surroundings are as obstacle free as possible. A commonly used metric for quantifying this criterion is the number of obstacles that could potentially hinder the operation of eVTOLs at each of the sites. Possible obstacles at Cologne Bonn Airport are the terminal buildings, parking garages *P2* and *P3*, the ventilation tower in the middle of parking garage *P1*, the hotel currently under construction and the road that provides access to the departure level of the two terminals. Smaller obstacles that are relatively easy to remove, such as lighting poles, are not taken into consideration.
- Noise impact on the adjacent buildings: The location is to be chosen in such a way that the number of people in the immediate vicinity of the vertiport who would be disturbed by eVTOL operations is kept to a minimum. This includes people working near the vertiport as well as customers and passengers. The most noise sensitive areas on the airport premises are the terminal buildings and the future hotel. The terminals are the workplace of many employees as well as an entry and exit point for passengers. The hotel is both a workplace and a sleeping place for customers, which is why it is also considered a noise sensitive area. This analysis did not take into consideration the current noise disturbance experienced at each location, as this information was not available. This criterion only represents the noise impact in the immediate vicinity of the vertiport, not the noise impact on neighbouring residential areas. Due to the vertical capabilities of eVTOLs, it is assumed that the specific location of the vertiport within the airport premises will not have a significant influence on which residential areas will be impacted by noise. This will rather be influenced by the departure and arrival routes defined for the vertiport.
- Expandability: the site must have areas in its vicinity that could be used for the expansion of the vertiport through the construction of additional FATOs, taxiways, stands and parking positions.
- Applicability: the time and financial costs to construct a vertiport at a certain location should be as low as possible.
- Strategic availability: The site should be available for possible development, taking into account the strategic plans of the Cologne Bonn Airport operator. The airport operator is currently examining infrastructure development possibilities at several locations for different purposes. The development of a vertiport at one of these locations would mean that the current plans would have either to be changed or completely reformulated. Furthermore, some locations at the airport have a strategic value for the airport operator, for which building a vertiport at one of these locations would also be suboptimal.

## Location analysis

### Selection of possible locations

**Fig. 2:**
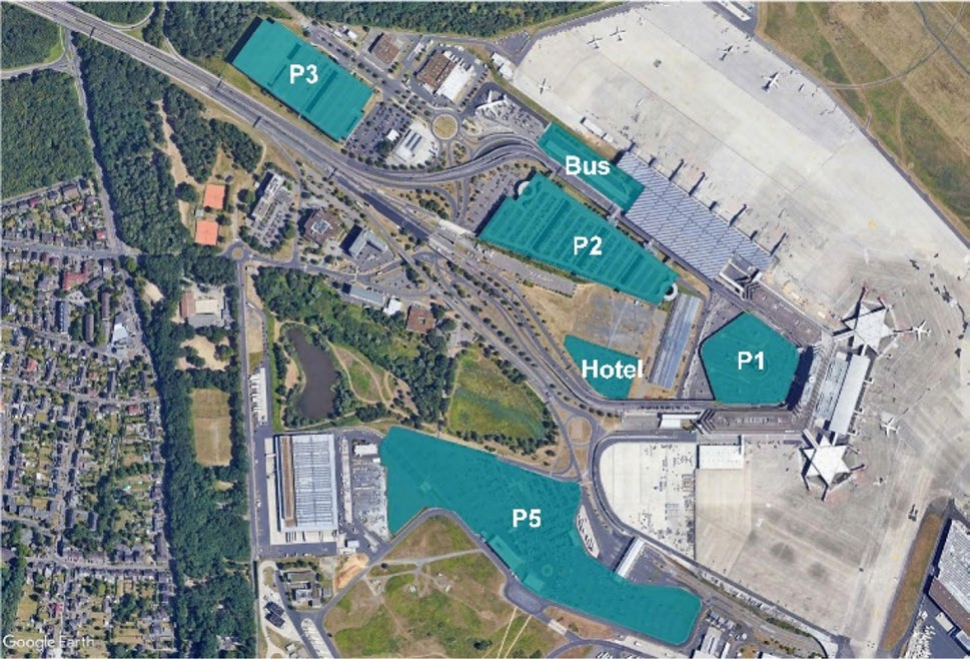
Possible locations for a vertiport at Cologne Bonn Airport

The first identification of possible sites for the development of a vertiport on the landside of the Cologne Bonn Airport is based on surface availability of the area as well as on the airport operator internal planning. The sites found to be most suitable can be seen in Fig. 2, marked by blue shading. The sites *P1*, *P2* and *P3* represent a parking garage each. The development of a vertiport on these sites would make use of the surface of the rooftop level of the respective car park building. The development of a vertiport on the *Bus* site, representing the existing long distance bus terminal, would require the construction of a structure on top of the bus terminal, in which eVTOLs could operate. A similar structure would have to be built on the *Hotel* site, which is a hotel currently under construction at the airport. At site *P5*, which is a single level car park that is currently mainly used by airport employees, a vertiport could be built directly on the existing surface, or on a structure built on top of the car park.

### Location evaluation

The evaluation of each possible location is performed based on the criteria described in “Location selection criteria”. The rating system is split into three categories:

-  + (3 Points): the criterion is satisfactorily fulfilled
- 0 (2 Points): the criterion is fulfilled to a limited extent
− (1 Point): the criterion is not satisfactorily fulfilled

For each criterion, the rating of a location is made on a relative basis between sites, not on an absolute basis. This means that the rating given to a particular site for any given criterion depends on the performance of the other sites considered on the same criterion. Furthermore, the final score of each location is achieved by simply adding the individual scores obtained in the multiple criteria. Consequently, every criterion considered has the same weight regarding the site selection. This approach is deemed reasonable in a first analysis of the possible locations. However, it is noted that in a further decision stage, each location’s pros and cons need to be analysed in more detail. Furthermore, the relative importance of each criterion may need to be taken into consideration by applying different weights in the evaluation process. The evaluation is presented in Table 2.

**Table 2:** Evaluation of the possible locations

Criteria	P1	P2	P3	P5	Bus	Hotel
Passenger accessibility	+	+	−	−	+	0
Obstacle clearance	−	0	+	+	−	+
Noise impact	−	0	+	+	0	−
Expandability	0	+	+	+	−	−
Applicability	−	0	+	0	−	−
Strategic availability	−	0	0	−	+	+
Total	9	14	15	13	11	11

#### Parking garage P1

With nine points, parking garage *P1* has the worst rating of all the locations considered. This is primarily due to the proximity of this location to Terminal 1, which borders it on three sides. Terminal 1 is therefore a major obstacle to eVTOL operations on *P1*. The site receives the worst rating in the noise pollution criterion, as the adjacent Terminal 1 is both an access and exit point for many passengers and customers and a workplace for a large number of employees. The poor strategic availability is due to the current plans for new construction measures in the parking garage. A necessary coordination of the projects would probably delay the development of a vertiport. The available space in *P1* is sufficient to allow a certain expansion of the vertiport, but it is smaller in comparison to other locations under consideration, for which it receives a medium rating in the criterion expandability.

#### Parking garage P2

The parking garage *P2* is located directly adjacent to Terminal 2 and received the second-best score with 14 points. It is within walking distance of Terminals 1 and 2, and has a significantly large area that could be used for the further development of a vertiport, for which it received the best rating in terms of the passenger accessibility and expandability criteria (3 points). In all other criteria, car park P2 received the medium score (2 points). Due to its proximity to the terminals, there are several obstacles that can hinder eVTOL operations, and both passengers and staff can be disturbed by the noise generated during operations (obstacle clearance and noise disturbance criteria). The structure of parking garage *P2* is currently in a relatively poor condition. For this reason, the structure would have to be completely renovated before a vertiport could be built on this site (applicability criterion). During the renovation process, it should also be ensured that the structure is able to absorb all loads that occur due to eVTOL operations. *P2* has currently a significant strategic value. Its roof level offers a considerable amount of parking spots, which represents a significant source of income for the airport (strategic availability). Although *P2* does not get the maximum score on many criteria, it is a very viable option for the development of a vertiport, its main advantage being the proximity to both terminals.

#### Parking garage P3

Parking garage *P3* is the best-rated location with a total of 15 points (one more than parking garage *P2*). It has the best rating in all criteria except passenger accessibility and strategic availability. Together with car park *P5*, it is the location furthest away from the airport terminals. The distance between *P3* and the terminals considerably reduces the attractiveness of an air taxi service to be offered at this location, as the travel time increases considerably due to the additional distance that would have to be covered. Although *P3* is within walking distance from both terminals (approximately 10 min), a shuttle service would have to be offered to make the air taxi service barrier-free. With regard to the strategic availability criterion, the roof level of the parking garage *P3* is currently a source of income for Cologne Bonn Airport (such as *P2*), for which this location receives the medium score in this criterion. In all other criteria, *P3* receives the maximum number of points. Due to its distance to the airport's major infrastructure, the operation of eVTOLs at this location is relatively unhindered, and the number of people disturbed by noise is also relatively low. The space available at *P3* is considerable, and the extension of a vertiport at this location would not constitute a problem. Finally, the structure of the parking garage *P3* is in relatively good condition. Before starting the construction of a vertiport at this location, it is only necessary to check whether the loads imposed on the structure due to the operation of eVTOLs can be absorbed without further intervention.

#### Parking garage P5

In third place, parking garage P5 reaches an overall score of 13 points. It is a relatively large area relatively distant from the terminals or any other larger structure. This results in the high score in the criteria of obstacle clearance, noise pollution, and expandability. The criteria that *P5* does not meet are passenger accessibility, feasibility, and strategic availability. Along with parking garage *P3*, *P5* is one of the farthest locations from the airport terminals, which is why it receives a poor score in the passenger accessibility criterion (1 point). The feasibility of this location depends on whether the vertiport should be built on the existing parking area or on a new elevated structure above the current parking lot. An elevated structure has the advantage of preserving the many parking spaces that would have to be removed if the vertiport were built at ground level. However, an elevated structure would take much longer to build and be more expensive. Because of this ambiguity, *P5* receives 2 points in the feasibility criterion. In terms of strategic availability, *P5* is a potential site for the development of different airport projects at the boundary between the landside and airside. For these reasons, parking garage *P5* receives a low score in the strategic availability criterion.

#### Bus

The development of a vertiport on a structure that would be built on the existing bus terminal next to Terminal 2 is probably the most complex project considered in this study. A completely new structure would have to be built here, integrating the entire vertiport including FATO, taxiways and stands on top of the existing bus terminal. The possible obstacles for eVTOL operations during approach and departure depend on the height of the structure on which the vertiport would be built. Terminal 2, parking garage *P2* and the elevated terminal driveway would likely be considered obstacles. In addition, due to the high financial investment required to build such a structure, it is assumed that the project will be dimensioned exactly for the initially needed capacity. For these reasons, the *Bus* location receives a poor rating in terms of the criteria obstacle clearance, expandability and applicability. The other criterion for which this location does not receive the full score is noise disturbance, as it is located directly next to Terminal 2 and above the long-distance bus terminal, which would affect a considerable number of passengers. The sum of the points obtained by the *Bus* location is merely 11 points.

#### Hotel

The hotel currently under construction is located between the elevated terminal driveway, the *P2* parking garage and the train station building. Although it is not as far from the terminal buildings as the *P3* and *P5* locations, it is further away than the *P1* and *P2* parking garages, for which only two points are awarded in the criterion passenger accessibility. It can be assumed that a vertiport built on the rooftop of the hotel would considerably disturb the hotel customers, even if it were only operated during daytime hours. For this reason, the location receives only one point in terms of noise disturbance. The space available for an extension of a vertiport on top of the hotel is extremely limited, which causes the score on the expandability criterion to be only one point. Furthermore, the hotel is already under construction, for which subsequently integrating a vertiport in this infrastructure would be extremely difficult, which results in a poor score on the applicability criterion. Concluding, the *Hotel* location also obtains a total of 11 points.

### Preferred locations

The two locations with the best score in the evaluation process described in “Location evaluation”, *P3* with 15 points and *P2* with 14 points, are considered the most suitable for the development of a vertiport on the landside of the Cologne Bonn Airport, and are further analysed in this section. The selection of these two locations is purely based on the evaluation process described in “Location evaluation”, including all the simplifications made. Therefore, the locations which were not selected for further analysis should not be directly discarded in a more detailed analysis at a further stage.

#### Parking garage P2

**Fig. 3:**
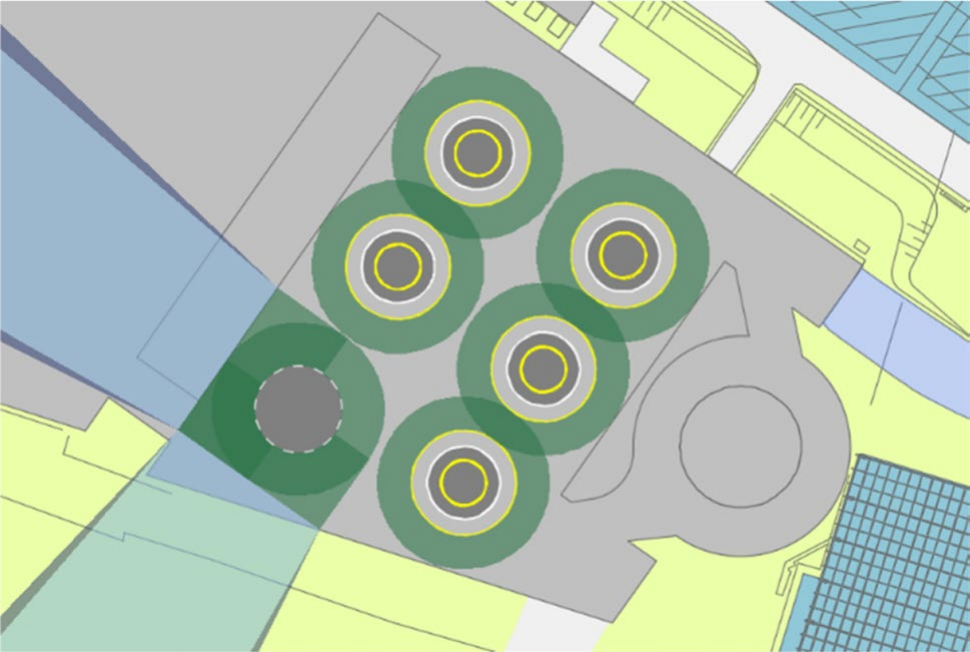
Example of a vertiport configuration in section 1 of parking garage P2

The parking garage *P2* is structurally split into four different sections (see Fig. 2), which increase in size from the southeast to the northwest direction (numbered in this study from one to four, beginning with the smallest and ending with the largest one). The analysis of *P2* starts with the smallest section of this parking garage (section 1). Figure 3 shows an example of a possible use of the area with a vertiport dimensioned for supporting eVTOLs up to 12 m largest overall dimension. A positioning of one FATO and five stands is only possible by overlapping the safety areas of each stand (these areas are shown in green in Fig. 3). The possible directions for the final approach and take-off manoeuvres of eVTOLs are reduced in such an arrangement due to the location of the stands around the FATO. Moreover, the availability of two of the five stands is dependent on the occupancy of the other stands. This significantly limits the flexibility with which these stands could be used, which ultimately leads to a reduction in capacity. Finally, the remaining space available for additional facilities, such as vehicle technical support or passenger handling, is very limited. For these reasons, a vertiport in section 1 of *P2* is deemed unfeasible.

“Cologne Bonn Airport scenario” of *P2* has significantly more available space. On this section, it is possible to place one landing pad and six stands, which corresponds to one additional stand to the minimum five needed (“Requirements on stands”). Additionally, there is sufficient remaining space on this section for support infrastructure such as passenger processing facilities and for technical support equipment. Since it is possible to set up a vertiport that meets the minimum requirements in the second section, “Vertiport infrastructure” and “Location analysis” must not be considered separately. It can be assumed that the necessary infrastructure could also be accommodated in those sections.

#### Parking garage P3

Similar to *P2*, parking garage *P3* is also structurally divided into different sections, which are numbered in this study from one to three in the same direction as the sections of *P2*. All the sections of *P3* have approximately the same area for which an initial vertiport could be built in any of the three sections (the remaining two sections would serve as available area to eventually expand the vertiport). In this study, the construction of an initial vertiport in section 1 is further analysed. Like *P2*, for a vertiport dimensioned to serve eVTOLs up to a largest overall dimension of 12 m, section 1 of *P3* has sufficient space for one FATO and at least six stands. The available space is also considered sufficient to accommodate the infrastructure required for passenger handling as well as technical support equipment.

#### Obstacle analysis

**Fig. 4:**
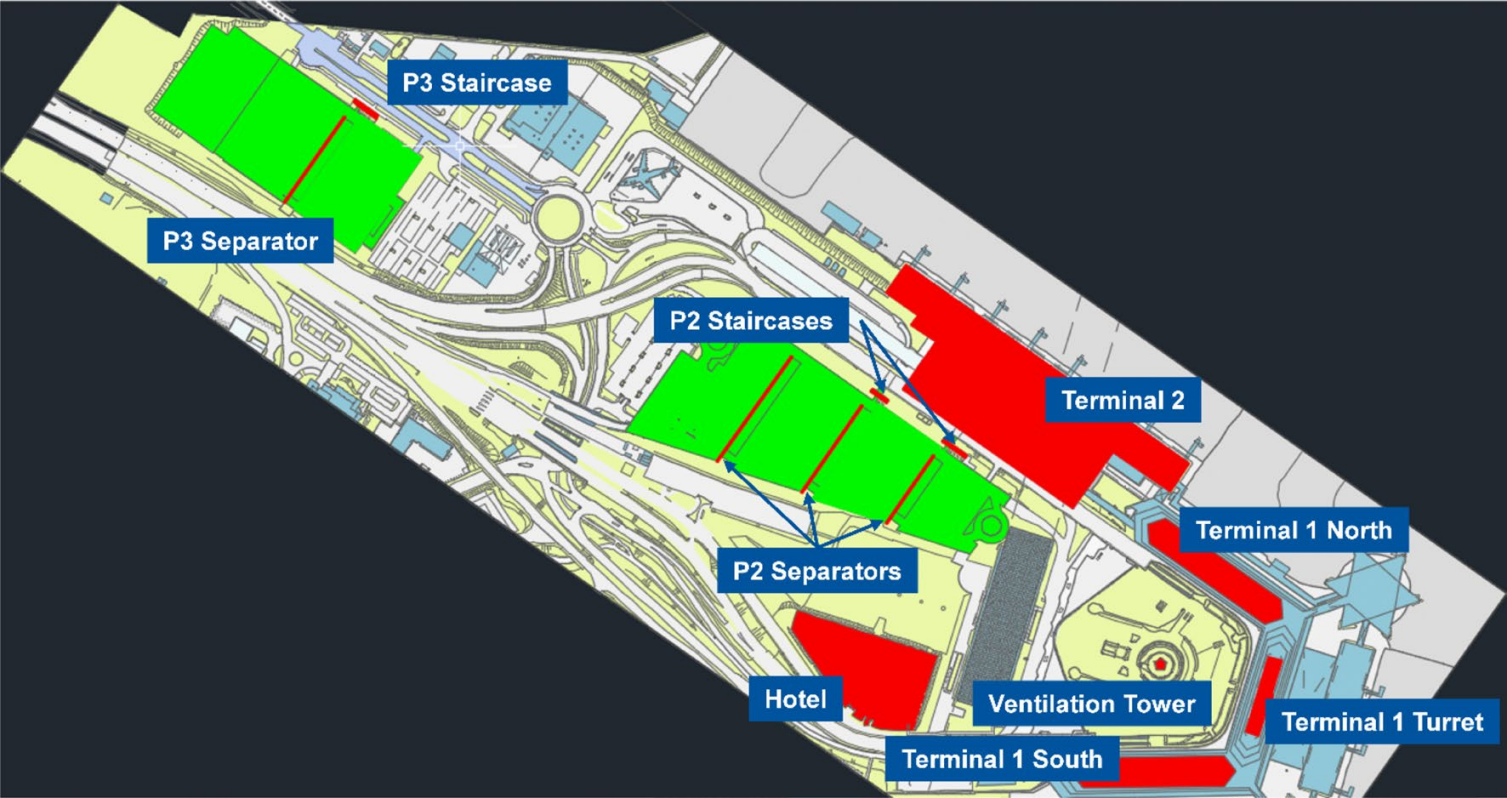
Possible obstacles for a vertiport at *P2* or *P3*

Besides the available area for the development of a vertiport, another important aspect to choose a given location is the obstacle environment around that location. The potential obstacles for a vertiport to be developed on one of the parking garages *P2* or *P3* are shown in Fig. 4. This includes all infrastructures at the airport that are higher than the two parking garages.

**Fig. 5:**
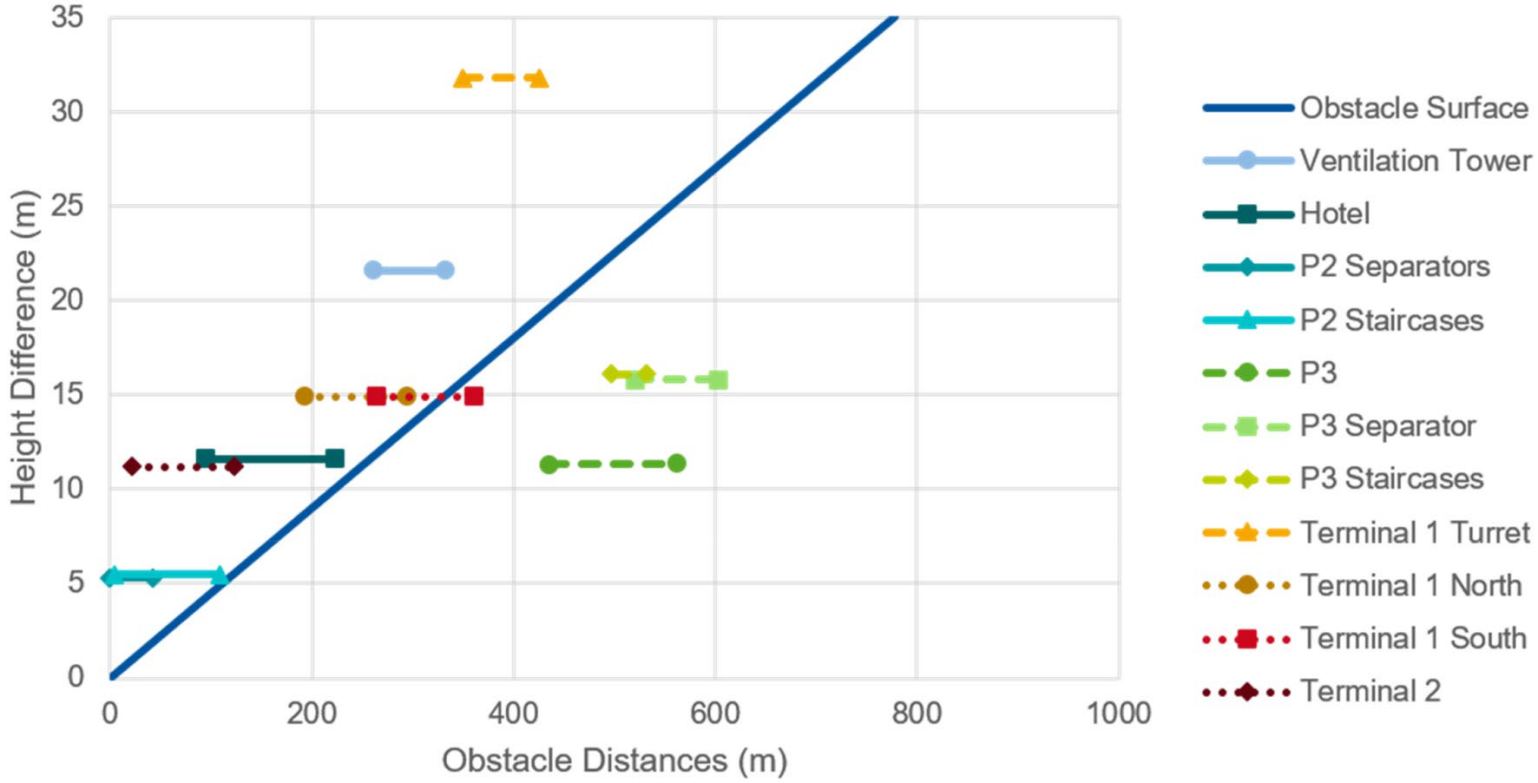
Obstacle analysis of *P2* section 2

**Fig. 6:**
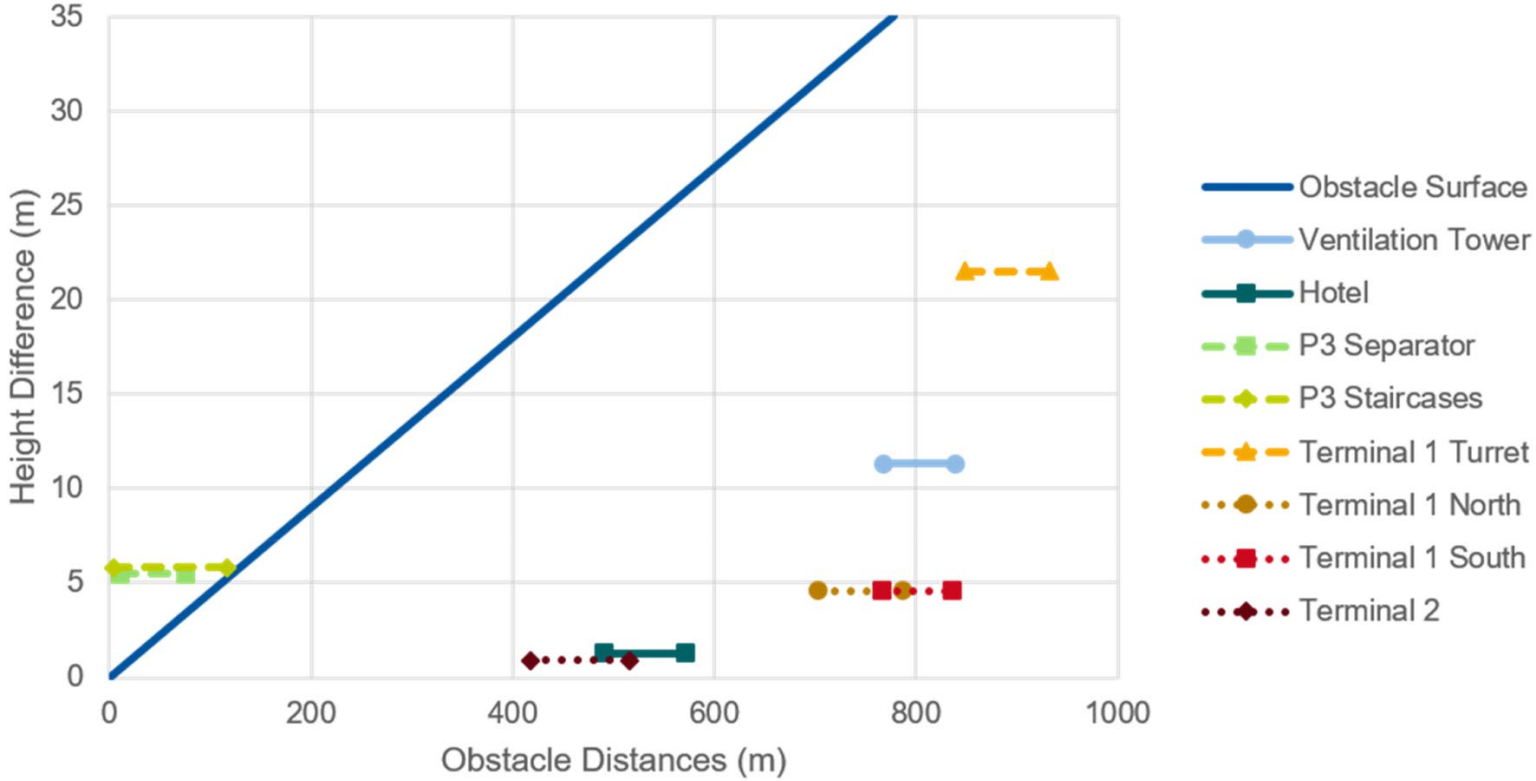
Obstacle analysis of P3 section 1

As mentioned in “Regulatory requirements”, this study assumes a slope of 4.5% for the definition of obstacle surfaces. With this assumption it can be calculated which infrastructures could be an obstacle for the operation of eVTOLs and intercept the obstacle clearance surfaces. Since an exact location of the FATO would only be determined in a further planning stage, the obstacle analysis is performed for a whole area, specifically section 2 of *P2* and section 1 of *P3*. Figures 5 and 6 show the obstacle surface and the heights and possible distances of the different potential obstacles for section 2 of *P2* and section 1 of *P3*, respectively. If an obstacle line is completely below the obstacle surface line, the structure is not considered an obstacle, regardless of the specific location of the FATO within each section. If an obstacle line is completely above the displayed obstacle surface line, the infrastructure is always an obstacle to a vertiport on that section regardless of the specific location of the FATO. Obstacle lines that cross the obstacle surface line represent building structures that may or may not be considered an obstacle, depending on the exact location of the FATO within each section.

The *P2* parking garage has a height of 72.6 m. From Fig. 5 it can be concluded that the obstacle surface of a vertiport in section 2 of *P2* would have to avoid the infrastructures of Terminal 2, Terminal 1, hotel and the ventilation tower. The separators of car park P2 would have to be removed, as well as some smaller infrastructures such as the lighting poles, which were not considered as a major infrastructure and therefore not included in the analysis.

Due to the higher elevation of *P3*, 83.9 m, there are fewer possible obstacles at this location. The separator and the staircases of *P3* are the only major obstacles that would need to be removed or avoided when defining obstacle clearance surfaces for a vertiport located at section 1 of *P3* (with the exception of lighter infrastructure such as lighting poles).

### Vertiport recommendation

Considering all topics analysed, section 2 of parking garage *P2* is currently the preferred location for the development of a vertiport at Cologne Bonn Airport. The integration of the air taxi service into the current transport network is of utmost importance for the success of this transportation method and influences both the acceptance and the attractiveness for the customers of such a service. For this reason, the proximity of *P2* to the two airport terminals is considered a major advantage over *P3*, which adds an average of 10 min walking time to the total travel time. Since section 1 of *P2* is too small to build a vertiport, as concluded in “Parking garage P2”, the preferred section of *P2* for the construction of a vertiport is section 2. There are no significant differences between the three largest sections of *P2*. Since section 2 is the closest section to the airport terminals where a vertiport could be built, it is currently the preferred section. The adjoining sections would then serve as areas for a possible extension of the vertiport, should this be necessary.

**Fig. 7:**
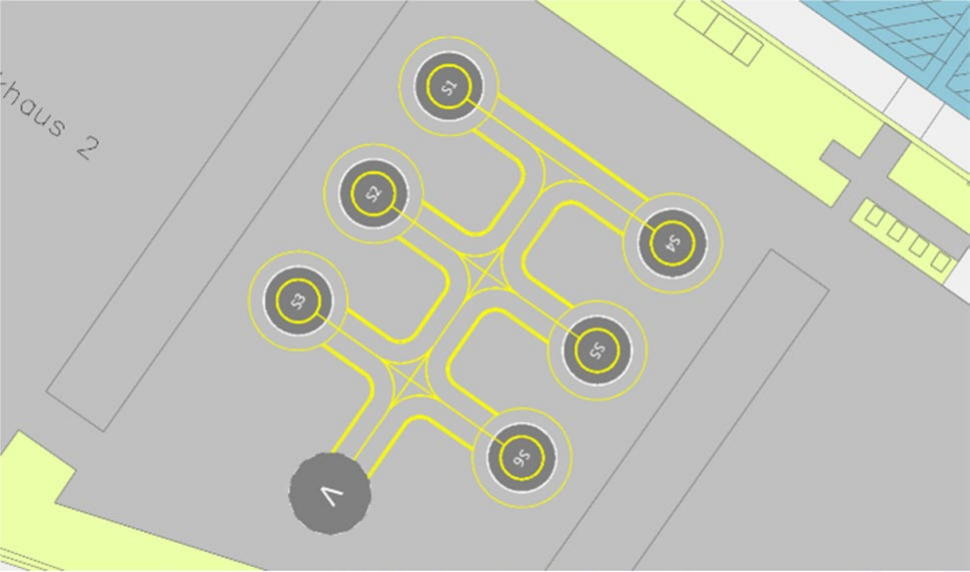
Recommended vertiport configuration at Cologne Bonn Airport

Figure 7 shows a vertiport with the FATO and stands configuration that is considered the most optimal for section 2 of *P2*. The vertiport is dimensioned for an eVTOL with 12 m maximum overall dimension. The FATO is located on the opposite side to the terminals, and the stands are placed in between. This minimizes the walking distance for passengers to reach each stand, and provides multiple flight paths for landing and take-off that would not need to overfly the vertiport itself. For this configuration, obstacle clearance surfaces could be defined for both the northwest and southeast directions (approx. 320° and 140° respectively), which is the same orientation as the airport’s main runway. In addition to removing the *P2* separators, the southeast obstacle clearance surface would have to bypass the infrastructures of Terminal 1, the ventilation tower and the hotel (achievable by introducing a turn in the obstacle clearance surface). The space available in section 2 of *P2* also ensures that the movement of passengers and eVTOLs can be safely separated (in this configuration, passengers could move along the edge of section 2 to reach the stands).

## Passenger processing

The following section presents the requirements and a concept for a terminal system and passenger processing components of an air taxi service. For this work, we focus on the processing system for departing passengers. The passenger processing system is based on the conventional passenger processing currently observed at any commercial airport. Typically, departure processing includes ticketing, check-in and baggage drop-off [16]. Furthermore, security screening constitutes a mandatory process for passengers. For international flights, passport control is mandatory as well. After clearing all process stations, passengers leave the terminal system by boarding the aircraft.

Regarding the required passenger processing system for an air taxi service at Cologne Bonn Airport, ticketing and check-in are expected to be mainly conducted through digital devices such as smartphones. However, a physical counter for providing information and basic services cannot be omitted. Since the maximum range of the considered eVTOLs is claimed to be 300 km [17] and the Cologne Bonn Airport is located at a distance of more than 300 km from any Non-Schengen country borders, it is assumed that only flights within the Schengen area will be operating from a vertiport at Cologne Bonn Airport. Thus, no passport control needs to be included in the terminal system.

Civil aviation security is regulated in Europe by the *Regulation (EC) No 300/2008* [18]. This document defines the requirement of access controls and passenger and cabin baggage screening and protection. Additionally, the *Commission Regulation (EU) No 1254/2009* sets criteria *“to allow Member States to derogate from the common basic standards on civil aviation security and to adopt alternative security measures”* [19]. The regulation applies to traffic of aircraft with a maximum take-off weight of less than 15 000 kg, which covers the eVTOLs considered in this study. For this traffic category, alternative security measures that provide an adequate level of protection on the basis of a local risk assessment may be adopted at the airport. Examples for general aviation without passenger security screening are aircraft movements starting at small airfields such as Aachen-Merzbrück in Germany, or existing air taxi services such as *BLADE* [20]. Therefore, the necessity of a passenger security screening needs to be clarified for the specific case of Cologne Bonn Airport and will in the end be determined by the corresponding regulatory authorities. Regardless of the necessity of security controls, an access control should be constructed to avoid non-authorized passengers or visitors walking onto the manoeuvring area of the vertiport due to safety and security reasons.

Additionally, considering eVTOL safety issues, the process of weight balancing is required prior to every individual flight. Thus, passenger and baggage weight need to be determined and documented in a corresponding load sheet. Having completed the passenger processing components, a waiting area is required for passengers, which are ready for boarding the next available eVTOL.

## Vertiport simulation

### Simulation method

**Fig. 8:**
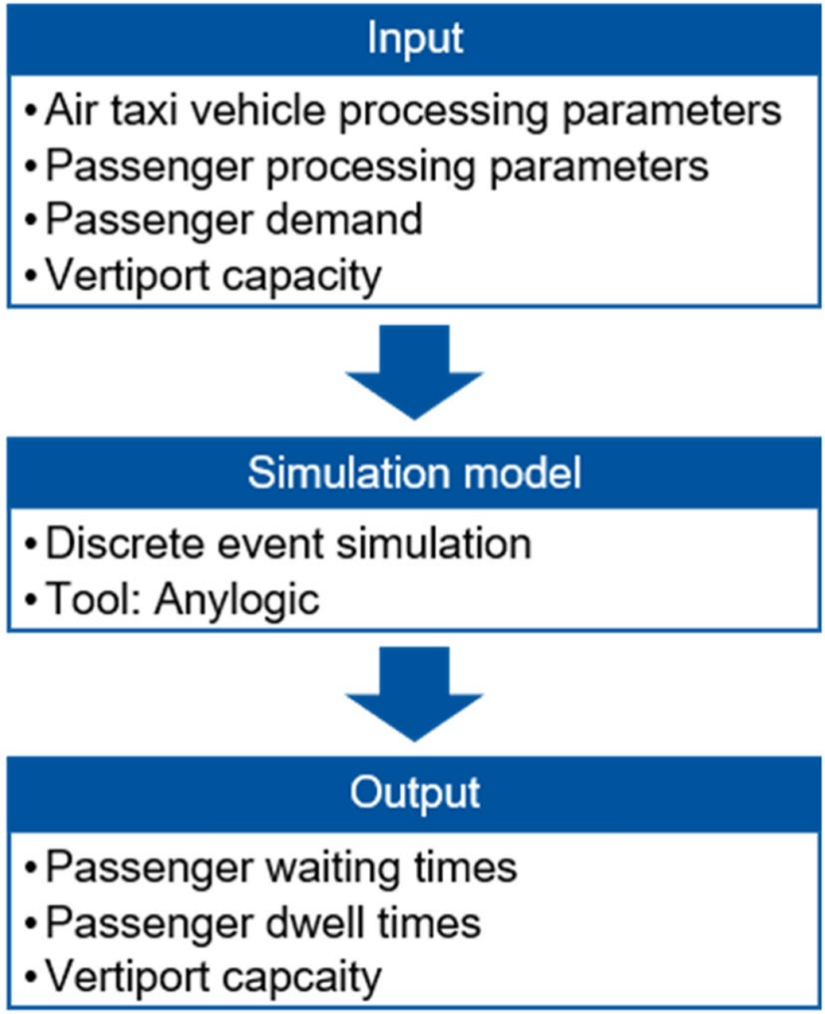
Simulative analysis approach

Having discussed the eVTOL and passenger processing infrastructural requirements for a vertiport, the final section of this paper addresses a first approach and results of a simulative analysis of this system. The discrete event simulation tool *Anylogic* is used to analyse the processing system as previously presented on microscopic level. The simulation focuses on departing passengers and illustrates operations during daytime (from 6 a.m. to 10 p.m.) assuming good weather conditions (VFR). The simulation model is created for the recommended vertiport configuration at Cologne Bonn Airport. Input parameters are determined according to the Cologne Bonn Airport scenario (“Cologne Bonn Airport scenario”). Therefore, the maximum eVTOL seating capacity accounts for four passengers. The main intended output parameter for this simulation is the vertiport’s capacity. However, applying the estimated passenger demand, passenger waiting times and passenger dwell times are examined as well. As mentioned before, the simulated vertiport infrastructure is considered as initial part of a vertiport at Cologne Bonn Airport and is not designed to handle the estimated passenger demand. The simulation approach is depicted in Fig. 8.

For the simulation, we assume a high utilization of the controlled airspace in the environment of the vertiport, which means that whenever the vertiport is available for another arrival, an eVTOL is ready for landing. This assumption enables to focus on the capacity performance of the vertiport. As mentioned in “Requirements on stands”, a separation of three minutes is considered between two eVTOL movements. To investigate the maximum possible eVTOL utilization, we assume that all passengers using the same eVTOL travel to the same destination. Passenger processing times for access control and baggage check are assumed to be uniformly distributed within the range of 5–20 s and 30–60 s, respectively.

### Simulation results and discussion

The simulation output values are presented in Table 3, averaged throughout one operational day. The results show high waiting times for passengers being allocated to an available eVTOL. These waiting times are significantly higher than the waiting times for access control and baggage check. We therefore identify the airside to be the bottleneck of the vertiport. The maximum throughput of the vertiport is 38 passengers per hour, assuming 4-seated eVTOLs. The number of FATOs and the controlled airspace limit the overall performance of the vertiport. The expansion of the vertiport by another FATO would only be effective along with an according expansion of the airspace capacity. Since this study is based on VFR operations, the airspace capacity problem needs to be overcome first. Overall, the simulation shows a high utilization of the considered vertiport resulting in high waiting times that would exclude air taxi services operating at this vertiport as a viable transportation service. This result was to be expected due to the limited design of the vertiport, which is suggested to constitute only the initial infrastructure element of a future vertiport at Cologne Bonn Airport.

**Table 3:** Simulation output parameters

Output	Value
Vertiport capacity	9.6 movements per hour
Passenger waiting time (access control)	0.00 h
Passenger waiting time (baggage check)	0.14 h
Passenger waiting time (available eVTOL)	2.04 h
Passenger dwell time	2.48 h

## Conclusion and further work

The aim of this work was to determine infrastructure requirements for an air taxi service and to transfer these requirements to the specific situation at Cologne Bonn Airport. Considering the existing legal framework, we identified legal requirements for the design of the vertiport. Additionally, further selection criteria specific for the situation at Cologne Bonn Airport were examined. These requirements were then applied to the Cologne Bonn Airport to assess possible vertiport locations.

The parking garage *P2* was found to be the preferred option for the initial development of a vertiport. The analysis of a passenger processing system for air taxi services provided an overview of mandatory process components such as access control, baggage check and boarding. As a final step, the simulation of passenger and eVTOL processing constituted a first analysis of the vertiport performance. The simulation indicated the high utilization of a vertiport even at low passenger changing rates (single-digit percentage) from conventional modes of transport at Cologne Bonn Airport. The bottleneck of the vertiport was found to be the controlled airspace at the vertiport, since we assume VFR operations for the air taxi service considered in this work and the number of movements significantly increases the workload for the air navigation service provider.

As the technical and legal framework for the eVTOLs and the vertiport infrastructure is subject to dynamic development, the steps taken within this work need to be iteratively repeated. Furthermore, the methodology used to determine the optimal location of an initial vertiport at Cologne Bonn Airport can be further used to obtain insights on the suitability of different locations at any given airport for the operation of eVTOLs (see Fig. 1). Financial aspects of an air taxi service as well as route network modelling constitute topics for further research. A detailed investigation of the potential passenger demand is required to design an expanded vertiport with more than one FATO that can handle the passenger demand at an adequate level of service. This infrastructure should be included in further simulation studies. Moreover, the approval process for an air taxi service and the vertiport infrastructure is not yet clarified. This approval process will most certainly include the consultation of all impacted stakeholders through air taxi operations, for which the noise impact of air taxi services needs to be further analysed.

## Data Availability

Not applicable.
